# Grafting of Amines on Ethanol-Extracted SBA-15 for CO_2_ Adsorption

**DOI:** 10.3390/ma6030981

**Published:** 2013-03-12

**Authors:** Yong Li, Nannan Sun, Lei Li, Ning Zhao, Fukui Xiao, Wei Wei, Yuhan Sun, Wei Huang

**Affiliations:** 1 State Key Laboratory of Coal Conversion, Institute of Coal Chemistry, Chinese Academy of Sciences, Taiyuan 030001, China; E-Mails: liyong@sxicc.ac.cn (Y.L.); nnsun@sxicc.ac.cn (N.S.); lilei@sxicc.ac.cn (L.L); zhaoning@sxicc.ac.cn (N.Z.); xiaofk@sxicc.ac.cn (F.X.); 2 Graduated University of Chinese Academy of Sciences, Beijing 100049, China; 3 Low Carbon Conversion Center, Shanghai Advanced Research Institute, Chinese Academy of Sciences, Shanghai 201203, China; 4 ShanXi Lu’an Mining Industry (group) Co., Ltd, Changzhi 064204, China

**Keywords:** amine, grafting, ethanol extraction, SBA-15, CO_2_ adsorption

## Abstract

SBA-15 prepared via ethanol extraction for template removing was grafted with three kinds of amine precursors (mono-, di-, tri-aminosilanes) to synthesis new CO_2_ adsorbents. The SBA-15 support and the obtained adsorbents were characterized by X-ray diffraction (XRD), small-angle X-ray scattering (SAXS), N_2_ adsorption/desorption, thermogravimetry (TG), elemental analysis, Fourier transform infrared (FTIR) spectrometry, scanning electron microscopy (SEM) and transmission electron microscopy (TEM). It was found that, except higher silanol density, the ethanol-extracted SBA-15 support possessed a more regular mesophase and thicker walls than traditionally calcined samples, leading to a good stability of the adsorbent under steam treatment. The adsorption capacity of different amine-grafted samples was found to be influenced by not only the surface amine density, but also their physiochemical properties. These observations provide important support for further studies of applying amine-grafted adsorbents in practical CO_2_ capture process.

## 1. Introduction

CO_2_, which is produced from burning of fossil fuels, including coal, oil and natural gas, is the most important contributor to global warming. As fossil fuels are considered to be the major energy source in the next few decades (IPCC special report, 2007), the exploration of effective ways to stabilize the atmospheric concentration of CO_2_ has become an urgent task for human beings. Among different strategies proposed for CO_2_ mitigation, such as utilization of renewable energies *etc.*, CO_2_ capture and sequestration (CCS) has been considered as almost the only one effective and efficient option that matches the current energy infrastructure in the short-midterm [1].

It was reported that the costs of CO_2_ separation from flue gas, which is the most important CO_2_ emission sources [2], accounts for approximately 70% of the total cost for the CCS system [1,3]. Therefore, it is very important to develop new approaches for low-cost and energy-saving CO_2_ separation. As an alternative to the traditional liquid amine absorption processes, CO_2_ adsorption by solid adsorbents is considered to be a promising way, due to its capability of avoiding the high energy cost for sorbent regeneration and corrosion problems, which are encountered during the amine absorption process [4].

A series of solid adsorbents, including zeolites [5,6,7], activated carbons [8,9,10], hydrotalcites [11,12,13], metal-organic frame (MOF) materials [14,15,16,17,18,19], metal-oxide [20,21,22,23,24] and amine modified porous silica [25,26,27,28,29,30,31,32,33,34,35,36,37,38,39,40,41,42] have been studied for the separation of CO_2_. Among these adsorbents, amine modified porous silica (especially mesoporous silicas) has received considerable research interest due to their high adsorption capacity and selectivity towards CO_2_.

Amine modified porous silica can be divided by two classes according to the different synthesis methods, namely impregnation and grafting. For the impregnated adsorbents, high adsorption capacity for CO_2_ can be achieved by loading large amount of amines onto the support. However, weak interaction between amines and supports leads to poor thermal stabilities of these materials, which spoiled their application in practical processes [36]. By contrast, the grafted ones are more stable, due to the formation of chemical bonds between the surface silanols of silica and the aminosilanes. Mesoporous silicas with different pore structures (e.g., MCM-n, SBA-n and MCF) have been employed to synthesize various amine-grafted adsorbents [26,27,28,31,42,43,44,45].

For these materials, the amount of amine involved in the final adsorbents, which is an important factor influencing adsorption capacity, is mainly determined by the silanol density on the silica surface. Thus, the silica supports with high surface silanol density are desirable for the synthesis of adsorbents with high adsorption capacity. For template-assistant mesoporous silicas, the template-removing method is an important factor determining their surface silanol density. One pioneering work [32] had investigated the influence of different template-removing methods (extraction and calcination) on the silanol density of SBA-15 and the consequent effect on amine grafting for the preparation of solid CO_2_ adsorbents; it was that more silanol groups were preserved through extraction rather than calcination, therefore, leading to higher CO_2_ uptake for the samples from ethanol extraction. However, only monoamine was grafted and studied in their study, and the resulting structure differences between these two kinds of SBA-15 remain unidentified. Besides, extraction through a certain amount of ethanol (60 mL in their study) could not guarantee the effective removing of the templates.

**Chart 1:**
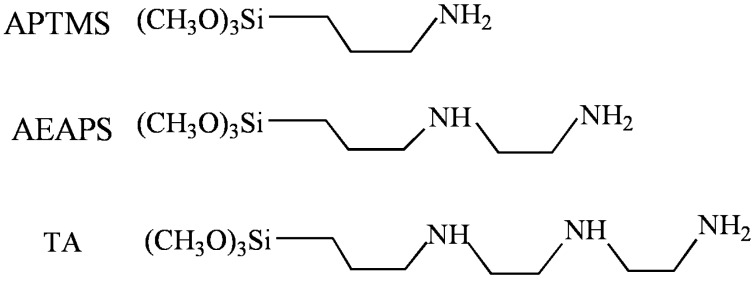
The molecular structures of the aminosilanes.

In this study, ethanol extraction through a Soxhlet extractor was used for the removal of the templates during the synthesis of SBA-15 support, which simplified the preparation procedure and improved the extracting efficiency. Except the aforementioned more surface silanol groups, the resulted SBA-15 possessed thicker walls, which were reported to play a decisive role to their hydrothermal stability [46], and the hydrothermal stability of these adsorbents is important for practical use, because steam-stripping was reported to be the most realistic way for the adsorbents regeneration [30]. More importantly, the effect of different amines was investigated by grafting three kinds of aminosilanes, namely 3-aminopropyltrimethoxysilane (APTMS), N-[3-(Trimethoxysilyl)propyl]ethylenediamine (AEAPS) and (3-trimethoxysilylpropyl)diethylenetriamine (TA) (Chart 1) onto the surface of SBA-15 support. The resulted adsorbents were characterized, evaluated and their stability under steam treatment was studied.

## 2. Results and Discussion

### 2.1. Characterization of SBA-15-c and SBA-15-ex Support

**Figure 1:**
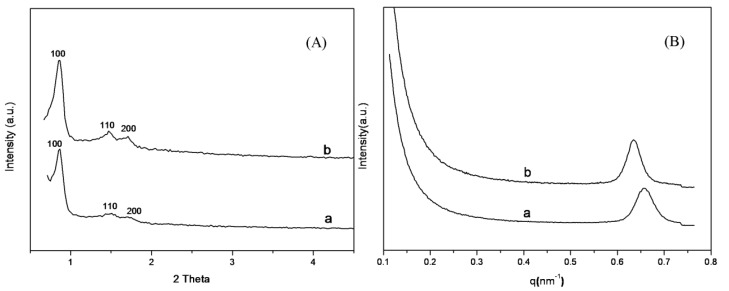
(**A**) X-ray diffraction (XRD); and (**B**) small-angle X-ray scattering (SAXS). Patterns of SBA-15-c and SBA-15-ex. a: SBA-15-c; b: SBA-15-ex.

The small angle XRD patterns of the SBA-15 support shown in Figure 1A exhibited well-resolved diffraction peak at 2θ = 0.8° and two weak peaks at 1.5° and 1.7°, due to (100), (110) and (200) reflections, respectively, indicating the existence of hexagonal mesophase within the samples [47]. The intensity of the diffraction peaks of SBA-15-ex was higher than that of SBA-15-c, which suggests that SBA-15-ex possessed a more ordered mesophase than SBA-15-c. The same trend could be observed on the small-angle X-ray scattering (SAXS) patterns of the SBA-15 samples when comparing the (100) plane diffraction peak intensity (Figure 1B). Besides, the diffraction peak of (100) plane of SBA-15-ex appeared at q = 0.633 nm^−1^, corresponding to a d spacing of 9.92 nm and a large unit-cell parameter (a_0_ = 11.46 nm), while for the calcined sample, the peak shifted slightly to a high q value (0.658 nm^−1^), indicating the decreasing of d spacing (9.54) and the unit-cell parameter (a_0_ = 11.01 nm). These results indicated that the high temperature during calcination would lead to the shrinkage of the SBA-15 skeleton and, thus, influencing the regularity of the formed mesophase. The more regular mesophase was not observed in previous work [32], which might be attributed to that extracting templates at boiling ethanol could also cause some collapse of pore structure and, thus, influence the regularity of the mesophase. While for the SBA-15-ex sample, these problems can be avoided owing to extracting at a much lower temperature.

**Figure 2:**
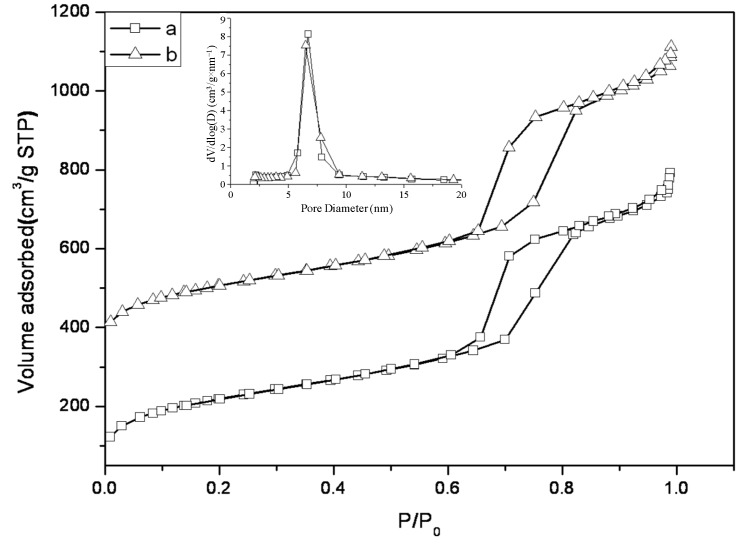
N_2_ adsorption–desorption isotherms and pore distribution curves of SBA-15. a: SBA-15-c; b: SBA-15-ex.

The surface area, pore size and pore volume of the SBA-15 supports were investigated by N_2_ adsorption/desorption. The isotherms and pore distribution curves are presented in Figure 2. Clearly, both samples exhibited typical type IV isotherms with a H1 hysteresis loops according to the IUPAC classification [48]; the difference between the two samples were negligible. The step increase at the relative pressure of 0.6 to 0.9 can be attributed to capillary condensation of N_2_ into the mesopores of the SBA-15 support. Correspondingly, the Barrett-Joyner-Halenda (BJH) pore size distribution curves (insert of Figure 2) were centered at 6.7 nm for SBA-15-c and 6.5 nm for SBA-15-ex, respectively, and a narrow distribution for both samples was observed. The results of texture properties were summarized in Table 1. SBA-15-ex showed that a surface area of 750 m^2^/g, which is quite similar to that of SBA-15-c (791 m^2^/g), indicated that the template was effectively removed by ethanol extraction from as synthesized SBA-15. Furthermore, thicker walls were observed on SBA-15-ex other than SBA-15-c, which could be mainly attributed to the lower temperature by extraction method leading to the less shrinkage of the silica framework [49].

**Figure 3:**
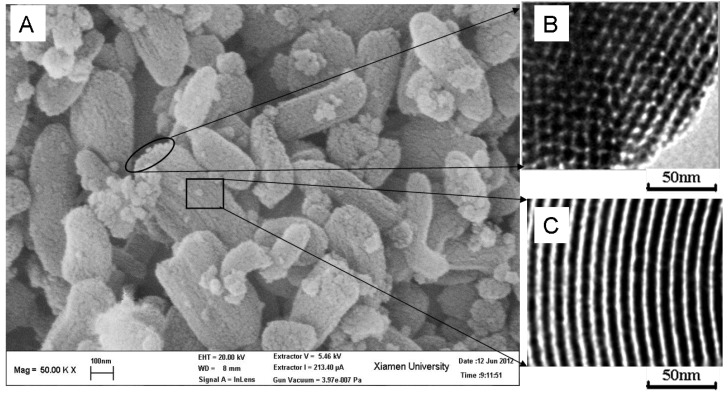
Scanning electron microscopy (SEM) and transmission electron microscopy (TEM) images of SBA-15-ex.

SEM and TEM images of SBA-15-ex are shown in Figure 3. Monodispersed rod-like particles with 500–800 nm in length and 200–500 nm in diameter could be observed in Figure 3A. This kind of morphology was reported previously [50]. The TEM images in Figure 3B,C confirm the ordered structure of the material; the cylindrical channels are arranged in an ordered hexagonal array, which is in accordance with the XRD and SAXS results.

**Table 1:** The textural parameters of SBA-15 and amine-grafted SBA-15.

Sample	S_BET _(m^2^/g)	V_Total _(cm^3^/g)	D_pore _(nm)	d spacing (nm)	a_0 _(nm)	W (nm)
SBA-15-c	791	1.23	6.7	9.54	11.01	4.3
SBA-15-ex	750	1.25	6.5	9.92	11.46	5.0
APTMS-SBA-15-ex	372	0.69	6.2	9.89	11.41	5.2
AEAPS-SBA-15-ex	410	0.67	6.0	9.88	11.41	5.4
TA-SBA-15-ex	286	0.50	5.7	9.89	11.41	5.7
APTMS-SBA-15-c	470	0.76	6.5	9.53	11.00	4.5

d spacing(d(100)) = 2*π/q*; a_0_ = $2\times \text{d}\left(100\right)/\sqrt{3}$. The wall thickness (W) was calculated as: W = a_0_ − D_pore_.

**Figure 4:**
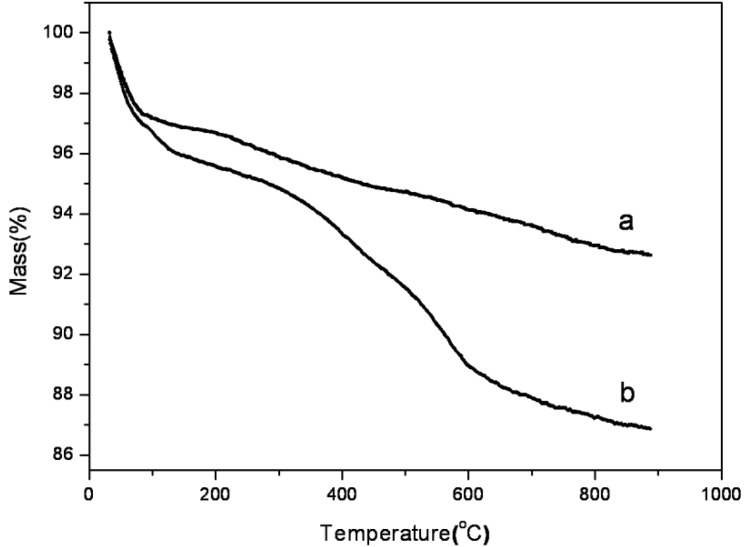
Thermogravimetric analysis (TGA) curves of a: SBA-15-c; b and SBA-15-ex.

The silanol densities on SBA-15-c and SBA-15-ex samples were analyzed by thermogravimetric analysis (TGA) [32]. As shown in Figure 4a, for SBA-15-c, the weight loss of 2.8% below 100 °C and the weight loss of 0.5% between 100 and 200 °C were attributed to the removal of the physically adsorbed and the chemically adsorbed water, respectively. The weight loss of 4.0% above 200 °C was attributed to the dehydroxylation by condensation of silanols. As for the SBA-15-ex sample (Figure 4b), the TGA curves was more complicated owing to the incomplete removal of the P123 template by ethanol extraction, that is, some of the P123 may be preserved in the pores of the final SBA-15-ex sample. Thus, at above 200 °C, the weight losses should be divided into two parts, namely the loss of the remaining P123 and the dehydroxylation by condensation of silanols. For pure P123, its degradation temperature range was reported in the range of 250 °C and 380 °C [32], while at the same temperature range, the weight losses of SBA-15-c and SBA-15-ex were 1.0% and 1.5%, respectively. The weight loss of SBA-15-ex at this temperature range was obviously lower than previous work (3.6%) [32], indicating the more complete removal of P123 in the present work. The maximum amount of any residual P123 could be deduced to be less than 0.5% (1.5%–1.0%), indicating that the weight loss of at least 8.1% (total: 8.6%) above 200 °C of SBA-15-ex could be attributed to the dehydroxylation of surface silanols and the coordinate silanol density was 9.0 mmol/g, which is two-times higher than that of SBA-15-c (4.4 mmol/g). These observations confirmed that more silanol groups could be preserved by using the ethanol extraction method than the traditional calcined method during the template removal process [32], which is naturally suitable for the grafting of amines. Thus, in this study, different kinds of amines were grafted on the SBA-15-ex for further research.

*The silanol density (ρ) was calculated as: ρ(mmol/g) = $\frac{2\text{m}}{{\text{M}}_{{\text{H}}_{2}\text{o}}}\;\times 1000$ while m(%) is the measured weight losses due to the dehydroxylation by condensation of silanols; ${\text{M}}_{{\text{H}}_{2}\text{o}}$ (mol/g) is the molar mass of water.

### 2.2. Characterization of Amine-Grafted SBA-15-ex

**Figure 5:**
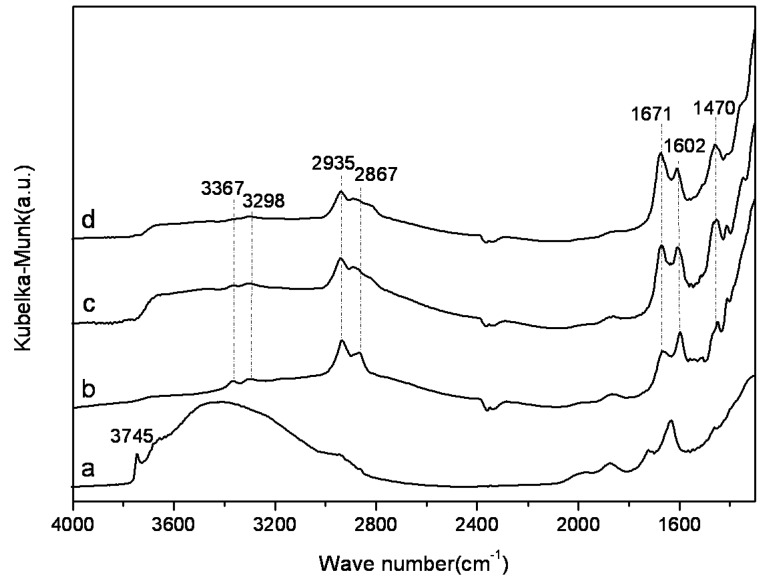
Diffuse reflectance infrared Fourier transform spectroscopy (DRIFTS) spectra of SBA-15-ex and amine-grafted SBA-15-ex. a: SBA-15-ex; b: APTMS-SBA-15-ex; c: AEAPS-SBA-15-ex; d: TA-SBA-15-ex.

Figure 5 shows the diffuse reflectance infrared Fourier transform spectroscopy (DRIFTS) spectra of SBA-15-ex and amine-grafted SBA-15-ex after surface cleaning at 100 °C for 1h under Ar flow. For SBA-15-ex, the sharp band at 3745 cm^−1^ could be attributed to the vibration of isolated hydroxyl group (Si-OH), and the overlapping peaks from 3000 to 3740 cm^−1^ were due to the internal hydroxyl group and hydrogen-bonded hydroxyl group [26]. After grafting, the intensity of these bands decreased, accompanied by the appearance of a series of new vibration peaks. Except the adsorption bands of the CH_2_ stretch (2935 cm^−1^, 2867 cm^−1^) and deformation (1471 cm^−1^), the bands at 3367, 3298 and 1602 cm^−1^ could be assigned to asymmetric NH_2_ stretch, symmetric NH_2_ stretch and NH_2_ deformation of hydrogen bonded amino group, respectively. The bands around 3300 cm^−1^ of AEAPS and TA grafted samples were not obvious, due to the overlap of NH stretch of secondary amine(-NH-) and primary amine(-NH_2_). These results confirmed the successfully grafting of amines onto the SBA-15-ex support.

**Figure 6:**
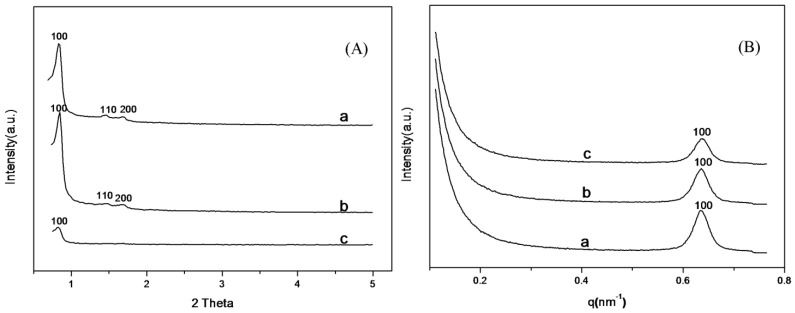
(**A**) XRD; and (**B**) SAXS patterns of amine-grafted SBA-15-ex. a: APTMS-SBA-15-ex; b: AEAPS-SBA-15-ex; c: TA-SBA-15-ex.

The small angle XRD patterns of amine-grafted SBA-15-ex are shown in Figure 6. The diffraction peak of (100) plane of hexagonal mesophase could be clearly observed, indicating that the mesoporous structure was basically preserved after grafting. The peak intensity of the samples decreased in the order of APTMS-SBA-15-ex > AEAPS-SBA-15-ex > TA-SBA-15-ex; especially for TA-SBA-15, the peak intensity of (100) plane decreased significantly, and the peak of (200) and (110) plane even disappeared. This result suggests that the regularity of the mesophase decreased with the increasing of group size of the grafted amines. A similar trend could be observed in the SAXS patterns of samples in Figure 6B. The peak intensity of (100) plane decreased following the same order, but the scattering vector (q) of the plane remained unchanged, revealing that although the regularity of mesophase decreased, the d spacing and unit-cell parameter kept constant, as shown in Table 1. This means the silica framework of SBA-15 was not influenced during the grafting process. The pore size of the amine-grafted samples also decreased in the following order of APTMS-SBA-15-ex > AEAPS-SBA-15-ex > TA-SBA-15-ex, which was also related with the increase of molecular sizes of different aminosilanes (Scheme I) as well and the wall thickness was found to be increased correspondingly.

**Figure 7:**
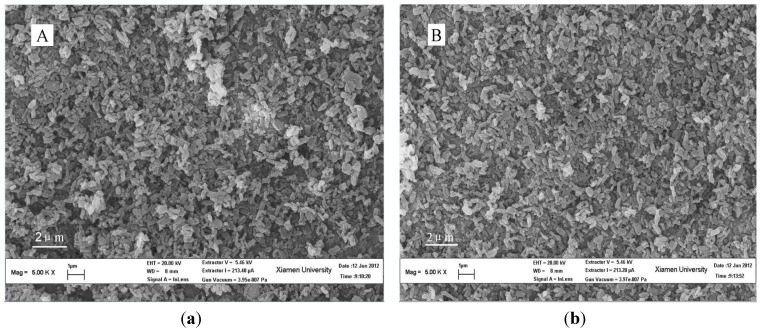

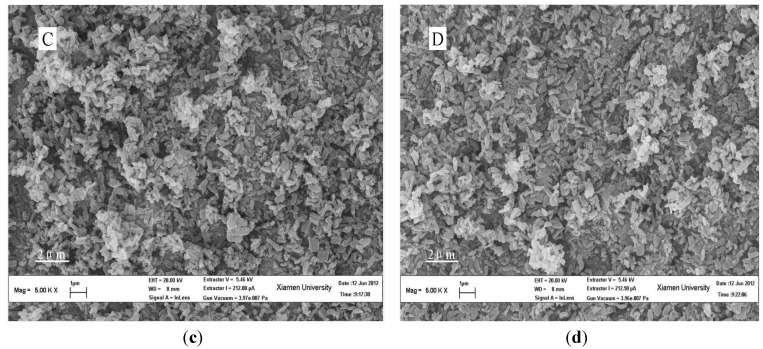
SEM images of SBA-15-ex and amine-grafted SBA-15-ex. (**A**) SBA-15-ex; (**B**) APTMS-SBA-15-ex; (**C**) AEAPS-SBA-15-ex; (**D**) TA-SBA-15-ex.

SEM images of the grafted samples are shown in Figure 7. No significant changes occurred for APTMS-SBA-15-ex (Figure 7B) comparing to SBA-15-ex (Figure 7A). While for the AEAPS- (Figure 7C) and TA- (Figure 7D) grafted samples, an aggregation of the aforementioned rod-like particles was observed. The possible explanation is that the introduction of organic component changed the surface affinity of SBA-15-ex; the larger organic groups are more flexible and prone to aggregate via the formation of hydrogen bonds and, thus, lead to partial aggregation of the nanosized particles. This aggregation may influence the diffusion of gases into the porous structure of the adsorbents.

### 2.3. Adsorption Properties of Amine-Grafted SBA-15

**Figure 8:**
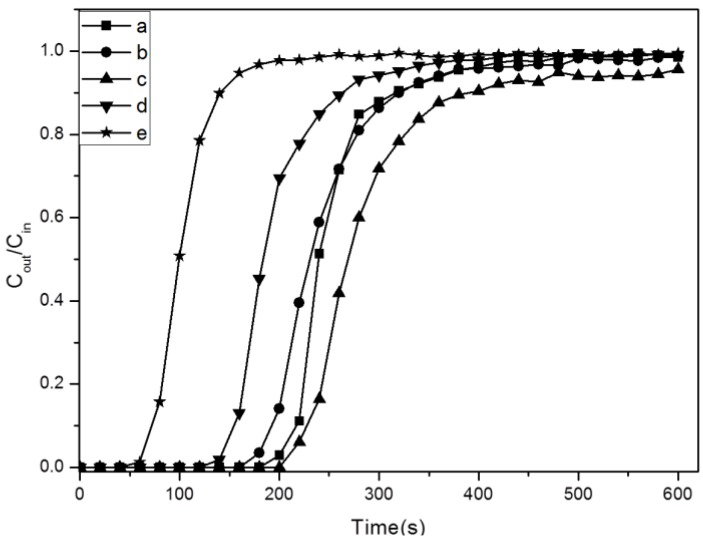
Breakthrough curves of CO_2_ adsorption at 25 °C. a: APTMS-SBA-15-ex; b: AEAPS-SBA-15-ex; c: TA-SBA-15-ex; d: APTMS-SBA-15-c; e: SBA-15-ex.

Figure 8 shows the breakthrough curves of CO_2_ adsorption at 25 °C. The breakthrough time of CO_2_ were in the following order: SBA-15-ex < AEAPS-SBA-15-ex < APTMS-SBA-15-ex < TA-SBA-15-ex. The slopes of the breakthrough curves were steep after breakthrough point, which means the equilibrium adsorption could be achieved rapidly. The slope of APTMS-grafted samples were steeper compared with other grafted samples, which may result from the aforementioned aggregation of the nanosized particles of AEAPS- and TA-grafted samples that limits the diffusion of CO_2_ and leads to a relatively slower kinetic.

**Figure 9:**
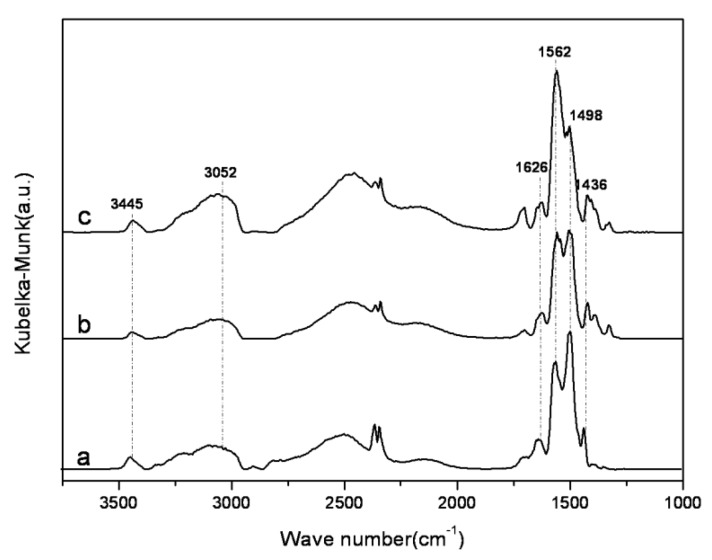
*In situ* DRIFTS spectra of amine-grafted SBA-15-ex. a: APTMS-SBA-15-ex; b: AEAPS-SBA-15-ex; c: TA-SBA-15-ex.

By integrating the breakthrough curves, the adsorption capacity of these samples was calculated and listed in Table 2. The amine contents analyzed by element analysis are also shown. It was found that the adsorption capacity of SBA-15-ex was significantly increased after grafting, indicating the clearly affirmative impact of amines for CO_2_ adsorption. And the amine content of APTMS-SBA-15-ex was obviously higher than APTMS-SBA-15-c (increasing of 50%); this is consistent with previous work [32], confirming that more amines were grafted on the support, due to the higher silanol density; consequently, the adsorption capacity was significantly increased. The adsorption capacity of 1.07 mmol/g was comparable to those adsorbents reported by other research groups, though the experiments conditions were different (see Table 3). However, it should be noted that the adsorption capacities of adsorbents were not fully consistent with the surface density of different amine-grafted samples. AEAPS-SBA-15-ex showed the lowest capacity of 0.99 mmol/g, and the highest capacity was achieved on TA-SBA-15-ex. The corresponding amine efficiency of all samples were 0.44, 0.29, 0.25 mol(CO_2_)/mol(N) for APTMS-SBA-15-ex, AEAPS-SBA-15-ex and TA-SBA-15-ex, respectively. According to the previous studies [26,27,51], the main species formed after adsorption of CO_2_ were alkylammonium carbamate under anhydrous conditions, which means two nitrogen atoms are required to adsorb one CO_2_ molecule, and the theoretical amine efficiency should be 0.5 mol(CO_2_)/mol(N). APTMS-grafted samples basically coordinated with this conclusion, but the deviation of AEAPS and TA-grafted ones were large. There are mainly two possible reasons accounting for these results. The first one is related to the previously studied particle aggregation, which may conceal some of amine groups from being reached by CO_2_. Another one is probably attributed to the different adsorption state of CO_2_ on different samples, which was further investigated by *in situ* DRIFTS study (see Figure 9).

**Table 2:** The amine content and the adsorption properties of the adsorbents.

Sample	Amine content (mmol/g)	N content (mmol/g)	Surface density (N atom nm^−2^)		Adsorption capacity (mmol/g)	Amine efficiency (mol(CO_2_)/mol(N))
SBA-15-ex	~0	~0	~0		0.21	-
APTMS-SBA-15-ex	2.44	2.44	1.94		1.07	0.44
AEAPS-SBA-15-ex	1.72	3.43	2.15		0.99	0.29
TA-SBA-15-ex	1.61	4.83	3.02		1.19	0.25
APTMS-SBA-15-c	1.62	1.62	1.31		0.75	0.46

**Table 3:** The adsorption data of amine-grafted adsorbents.

Adsorbents	Adsorption capacity (mmol/g)	Experimental conditions temperature/CO_2_ partial pressure	Evaluation method	Reference
APTMS -SBA-15	0.66	333 K/15 kPa	Flow method	[26]
APTES-SBA-15	1.5	298 K/10 kPa	TGA	[43]
APTMS-SBA-15	1.6	298 K/15 kPa	TGA	[32]
APTES-SBA-12	1.02	298 K/10 kPa	TGA	[43]
AEAPS-SBA-16	0.73	333 K/15 kPa	TGA	[28]
TRI-PE-MCM-41	2.05	298 K/5 kPa	Flow method	[42]
APTES-MCM-48	1.68	298 K/101 kPa	Static method	[52]
HA-SBA-15	1.00	298 K/10 kPa	Flow method	[53]

**Chart 2:**
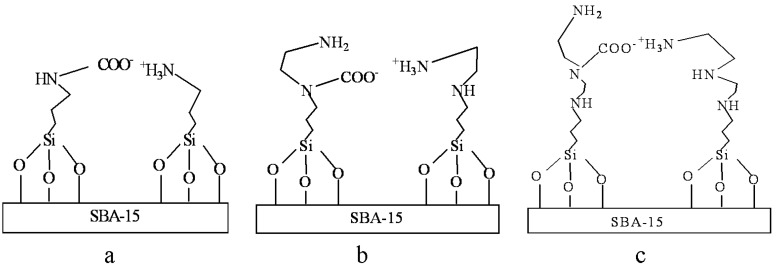
Possible CO_2_ adsorption species on different amine-grafted SBA-15. (**a**) APTMS-SBA-15-ex; (**b**) AEAPS-SBA-15-ex; (**c**) TA-SBA-15-ex.

Figure 9 shows the *in situ* DRIFTS spectra of the amine-grafted SBA-15-ex samples. The vibration peaks at 3445, 3052 and 1626 cm^−1^, which could be assigned to N-H stretch in RNHCOO^−^, N-H stretch in RNH_3_^+^ and N-H deformation in RNH_3_^+^ [51], respectively, suggesting the formation of alkylammonium carbamate [26]. Moreover, the sharp bands at 1,562 and 1,436 cm^−1 ^ could be attributed to the O-C-O asymmetric and symmetric stretching of COO- in alkyl carbamate anion. These bands are consistent with previous studies [26,27,51], indicating that the formation of alkylammonium carbamate. Furthermore, it is interesting to note that the relative intensity of the band at 1498 cm^−1^, which was due to N-H bending vibration of RNHCOO^−^ [54], decreased following the order: APTMS-SBA-15-ex > AEAPS-SBA-15-ex > TA-SBA-15-ex, indicating the decreasing numbers of RNHCOO^−^ groups following the same order. Thus, it could be deduced that, for AEAPS- and TA-grafted samples, different kinds of alkylammonium carbamates were formed, as shown in Chart 2. When one of the amine groups in the middle of molecular chain was used to adsorb CO_2_ with one terminal amine group, the other amine groups would be hardly available for CO_2_ adsorption, due to the steric hindrance of the chain. While for APTMS-grafted samples, higher utilizing efficiency of amine could be realized, due to the absence of the steric effect.

### 2.4. The Stability of Amine-Grafted SBA-15 after Steam Treatment

**Figure 10:**
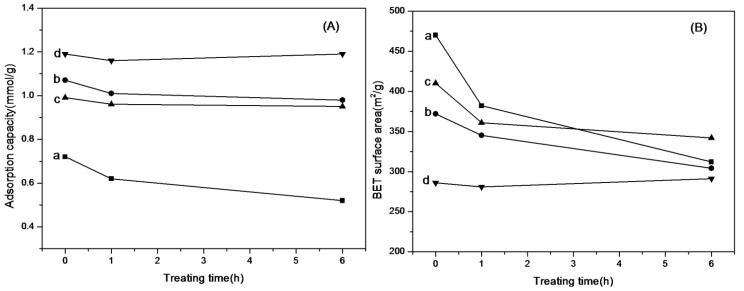

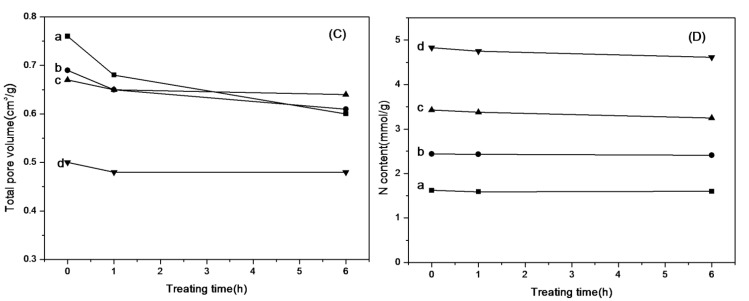
Comparison of (**A**) adsorption capacity; (**B**) Brunauer–Emmett–Teller (BET) surface area; (**C**) total pore volume; and (**D**) N contents of amine-grafted SBA-15 before and after steam treatment. a: APTMS-SBA-15-c; b: APTMS-SBA-15-ex; c: AEAPS-SBA-15-ex; d: TA-SBA-15-ex.

As the steam-stripping way was considered to be the most realistic way for adsorbents regeneration for CO_2_ capture, the stabilities of amine-grafted SBA-15 after steam treatment were evaluated and their physiochemical properties were also characterized, as shown in Figure 10. It can be seen that the adsorption capacity of the adsorbents (see Figure 10A) were all decreased after steam treatment, but the decreasing of amine-grafted SBA-15-ex was much less than APTMS-SBA-15-c. Besides, the N contents of all samples kept nearly constant during this process (see Figure 10D), indicating that little amines were removed from the adsorbents by steam treatment, and thus, the decreasing of adsorption capacity could mainly be attributed to the collapse or destruction of pore structure. The surface area of APTMS-SBA-15-c decreased approximately 20% after steam treatment for 1 h, and then another decrease of 15% was observed in the next 5 h, with a slower decreasing rate (see Figure 10B). While for APTMS-SBA-15-ex, the decrease of surface area was only 10% upon steam treatment for 1h, and the total decrease was less than 20%. The trends obtained from total pore volume were similar (see Figure 10C). Obviously the thicker walls of amine-grafted SBA-15-ex lead to a better pore structure stability, and consequently, the adsorption capacity of adsorbents was better maintained. Furthermore, it is interesting to note that the stability of adsorbents under steam treatment was different with different grafted amines; the adsorption capacities of AEAPS- and TA-grafted samples were not influenced remarkably during this treatment. Although the surface areas and pore volumes of AEAPS- and TA-grafted samples also suffered from some degradation at the first 1 h, they kept nearly unchanged during treatment from 1 to 6 h, especially for TA-SBA-15-ex. Reminding of the previously mentioned molecule size order, the possible explanation could be that the larger size of surface organic groups of the samples partly prevents the steam from being contacted with the silica framework and, thus, leads to less hydrolysis of Si-O-Si bond, which subsequently causes less collapse of pore structures.

## 3. Experimental Section

### 3.1. Preparation of SBA-15 Support

SBA-15 was synthesized according to the procedure reported by Zhao *et al*. [47]. Typically, 4.0 g of Pluronic P123 (EO_20_PO_7__0_EO_20_, Aldrich) was dissolved in 125 mL of 2 M HCl with stirring at 40 °C. Then, 8.5 g of tetraethyl orthosilicate (TEOS, Acros) was added into the above solution and further stirred at 40 °C. After 20 h, the mixture was then autoclaved and aged at 90 °C for 24 h without stirring. The obtained white solid product was then collected by filtration, washed with deionized water and dried in air at 80 °C for 24 h.

### 3.2. Removal of Template (P123) from As-Synthesized SBA-15

The template was removed by either calcination or ethanol extraction. In the calcination process, the as-synthesized SBA-15 was placed in a quartz boat and heated at 550 °C for 6 h in air. The obtained SBA-15 was denoted as SBA-15-c. For ethanol extraction, the as-synthesized SBA-15 (2.5 g) was placed in a Soxhlet extractor and extracted by 150 mL of ethanol (99.5%) for 20 h. The SBA-15 obtained was denoted as SBA-15-ex.

### 3.3. Grafting of Aminosilanes on SBA-15

Samples of amine-grafted SBA-15 were prepared by the following procedure: 2.5 g of SBA-15-c or SBA-15-ex; and, the calculated amount of aminosilanes were added into 150 mL of toluene. The mixture was stirred and refluxed at 80 °C for 12 h. The resulting amine-grafted SBA-15 was then placed in a Soxhlet extractor with 150 mL of toluene and extracted for 20 h to remove the unreacted aminosilanes, ensuring that all the amines remain on the surface were grafted rather than physically adhered. The extracted samples were vacuum dried at 80 °C overnight.

### 3.4. Characterizations

Powder X-ray diffraction (XRD) patterns were recorded on a Panalytical X’Pert Pro X-ray diffractometer with Cu Kα radiation, in the step mode (0.0167°, 10 s) in the range of 0.5 < 2θ < 5°. Small angle X-ray scattering (SAXS) was performed at Shanghai Synchrotron Radiation Facility (SSRF). The wavelength of the X-ray was 0.124 nm, and the distance between the sample and detector was 5 m. N_2_ adsorption-desorption was carried out at liquid nitrogen temperature (−196 °C), using a Micromeritics Tristar 3000 instrument. Samples were degassed at 200 °C overnight prior to testing. The surface areas of the samples were determined by the BET (Brunauer–Emmett–Teller) method [55], and pore size distribution was calculated from the desorption branch by using the BJH model [56]. The morphology of the samples was observed by a Hitachi S-4800 scanning electron microscope (SEM) operated at 20.00 kV. Thermal gravimetric (TG) analysis was carried out with a NETZSCH STA 409 PC Luxx instrument under N_2_ flow (50 mL/min, 99.99%) from 35 °C to 850 °C with a heating rate of 5 °C/min.

*In situ* diffuse-reflectance flourier transform IR study (*in situ* DRIFTS) of samples was performed on a Nicolet 550 FT-IR spectrometer equipped with an mercury cadmium telluride (MCT) detector. The samples were heated in an Ar flow at 100 °C for 1 h prior to analysis in order to remove adsorbed CO_2_ and H_2_O. The DRIFTS spectra before adsorption of CO_2_ were recorded at 25 °C in an Ar (50 mL/min, 99.99%) flow (resolution: 4 cm^−1^, 100 scans). Adsorption was carried out by switching the inert gas to CO_2_ (50 mL/min, 99.99%) for 5 min; then, Ar was switched back to remove any residual physical adsorbed CO_2_, and the spectra of *in situ* DRIFTS were collected with pretreated adsorbents as the background.

### 3.5. CO_2_ Capture Behavior

The CO_2_ adsorption experiments were performed in a U-shaped quartz tube (inner diameter, 5 mm). The CO_2_ concentration in the outlet was monitored by an on-line gas analyzer (Vaisala, Vantaa, Finland) with a sampling rate of every 20 s.

In a typical experiment, 0.7 g of the adsorbent was pretreated under an Ar (99.99%) flow of 60 mL/min at 100 °C for 2h, after which the system was cooled to 25 °C and kept at this temperature. The argon flow was then switched to a 10%CO_2_-90%N_2_ mixture at a flow rate of 60 mL/min to allow adsorption. According to the mass balance of CO_2_, the adsorption capacities could be determined as follows [23]:

(1) $$q=\frac{Q\left(t_{s}-t_{0}\right)C_{in}}{22.4W}$$

(2) $$t_{s}=\int_{0}^{t}(1-\frac{C_{out}}{C_{in}})dt$$

where t_s_ is the mean residence time (s); t_0_ is the residence time of the empty bed; C_in_ and C_out_ are the inlet and outlet CO_2_ concentration, respectively; q is the equilibrium absorption capacity of CO_2_ (mmol/g); t is the absorption time (s); Q is the volumetric feed flow rate (mL/s) at standard temperature and pressure (1 atm and 0 °C); and W is the weight of the adsorbent (g); t_0_ is measured by using the inert gas [57].

### 3.6. Steam Treatment of the Adsorbents

A certain amount of the adsorbents was packed by a piece of filter paper and fixed in the middle of a steamer to prevent the adsorbents from being contacted with liquid water. Then water was added into the steamer and heated to 100 °C to regenerate steam flowing through the adsorbents for a given duration; after then, the treated adsorbents were taken out and dried under a vacuum at 80 °C overnight for further evaluation and characterization.

## 4. Conclusions

The ethanol-extracted SBA-15 was synthesized through removing the templates by a Soxhlet extractor. Except previously reported higher surface silanol density, the ordered mesophase remains unchanged. At the same time, owing to the absence of thermal treatment, the crystalline pore wall suffered less shrinkage; therefore, a thicker wall of the resulted SBA-15 was obtained, which is beneficial for the structural stability. Three kinds of amines were successfully grafted onto the SBA-15 support; a slight degradation of mesostructure and aggregation of nanosized particles was observed. The adsorption capacity of APTMS-grafted SBA-15-ex was found to be obviously higher than that of APTMS-SBA-15-c, due to the higher surface amine density. However, the adsorption capacities of different amine-grafted samples were not fully consistent with the surface amine density, which was found to be related to the aggregation of nanosized particles and the different adsorption state (alkylammonium carbamate) of CO_2_. Good stability under steam treatment for amine-grafted SBA-15-ex was observed, which could be mainly attributed to the thicker walls. In addition, the stability of adsorbents under steam treatment was also found to be correlated with the different type of grafted amines; namely, bigger grafted groups lead to a better stability.
